# Glucose Metabolism, Neural Cell Senescence and Alzheimer’s Disease

**DOI:** 10.3390/ijms23084351

**Published:** 2022-04-14

**Authors:** Qianqian Wang, Linyan Duan, Xingfan Li, Yifu Wang, Wenna Guo, Fangxia Guan, Shanshan Ma

**Affiliations:** 1 School of Life Sciences, Zhengzhou University, Zhengzhou 450001, China; wqq970123@163.com (Q.W.); duanlinyan4881@163.com (L.D.); lixingfan0929@163.com (X.L.); wyf0247@163.com (Y.W.); guowenna@zzu.edu.cn (W.G.); 2 Institute of Neuroscience, Zhengzhou University, Zhengzhou 450052, China; 3 NHC Key Laboratory of Birth Defects Prevention, Henan Institute of Reproduction Health Science and Technology, Zhengzhou 450002, China

**Keywords:** glucose metabolism, neural cell senescence, Alzheimer’s disease, metabolic reprogramming, therapeutics

## Abstract

Alzheimer’s disease (AD), an elderly neurodegenerative disorder with a high incidence and progressive memory decline, is one of the most expensive, lethal, and burdening diseases. To date, the pathogenesis of AD has not been fully illustrated. Emerging studies have revealed that cellular senescence and abnormal glucose metabolism in the brain are the early hallmarks of AD. Moreover, cellular senescence and glucose metabolism disturbance in the brain of AD patients may precede amyloid-β deposition or Tau protein phosphorylation. Thus, metabolic reprogramming targeting senescent microglia and astrocytes may be a novel strategy for AD intervention and treatment. Here, we recapitulate the relationships between neural cell senescence and abnormal glucose metabolism (e.g., insulin signaling, glucose and lactate metabolism) in AD. We then discuss the potential perspective of metabolic reprogramming towards an AD intervention, providing a theoretical basis for the further exploration of the pathogenesis of and therapeutic approach toward AD.

## 1. Introduction

**Figure 1:**
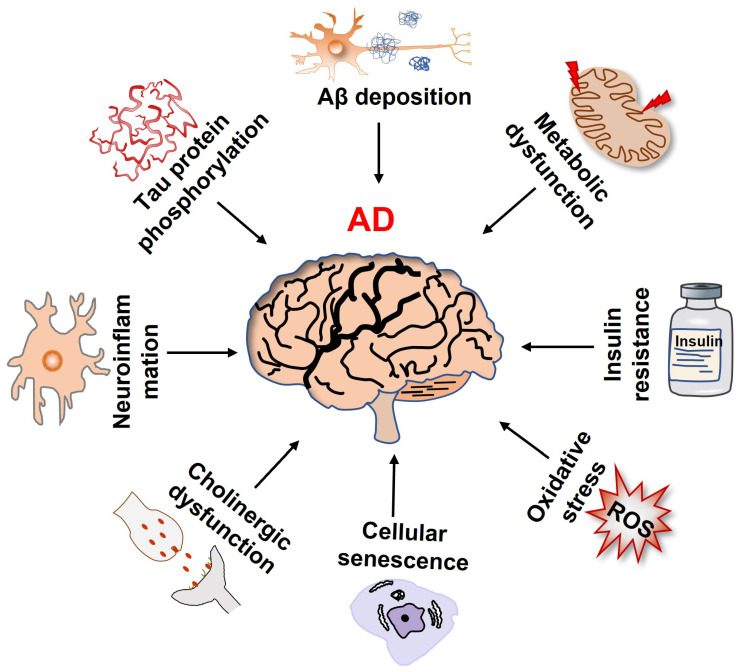
Multiple factors that contribute to the onset and progression of Alzheimer’s disease.

Alzheimer’s disease (AD), an elderly neurodegenerative disorder with a high incidence and progressive memory decline, is the major cause of dementia and is often accompanied by psychiatric symptoms [1]. Currently, there are no effective drugs to prevent and treat AD, which has become one of the most expensive, lethal, and burdening diseases of this century [2]. The pathological features of AD mainly include amyloid-β (Aβ) deposition and neurofibrillary tangles (NFTs) caused by the hyperphosphorylation of Tau protein. Several theories underlying the pathogenesis of AD have been posited (Figure 1), such as the amyloid protein cascade, Tau protein hyperphosphorylation, cholinergic dysfunction, oxidative stress, and neuroinflammation [3,4,5,6]. Currently, accumulating studies have shown that abnormal glucose metabolism in the brain is an early event before the pathological features of Aβ deposition. Thus, metabolic abnormalities are also considered to be the driving factors and hallmarks of AD [7,8]. The gradual decline in metabolic efficiency during aging renders neurons more vulnerable to toxic damage [9]. In addition, cellular senescence is also considered to be the main cause of cognition decline in AD. Although the relationship between metabolic imbalances, cell senescence, and AD pathogenesis has not been thoroughly elucidated, glucose metabolism in neural senescence paves a new way for a therapeutic strategy of AD [10]. In this paper, we undertook a brief review of the research progress in glucose metabolism, neural cell senescence, and AD, and discussed the potential application of glucose metabolic reprogramming for AD prevention, which may provide a theoretical basis for further understanding the pathogenesis and treatment of AD.

## 2. Neural Cell Senescence and AD

Aging is the most common risk factor of AD [11]. And, aging brain is caused by the accumulation of neural cell senescence, which is mainly manifested by mitochondrial dysfunction, impaired energy metabolism, senescence-associated secretory phenotypes (SASPs), and chronic neuroinflammation [12]. Senescent neural cells, including neurons, astrocytes, microglia, and oligodendrocytes, have been widely detected in the brain of AD mice and patients [13,14,15]. Accumulating evidence has indicated that cellular senescence plays an important role in the pathogenesis of AD [16,17]. An AD mouse model with an overexpressed human Tau gene accumulated p16Ink4a-positive senescent astrocytes and microglia [18]; astrocytes near NFTs displayed distinct senescent phenotypes in AD brains [19]. Aβ-aggravated neuronal senescence and cognition deficits were discovered in a 5×FAD mouse model of AD [20]. The clearance of senescent cells either pharmacologically or genetically could prevent Aβ deposition, Tau pathology, and cognitive decline in AD mice [18,21,22]. Although the cause and effect relationship between cellular senescence and AD remains controversial, growing studies imply that Aβ triggers neural cell senescence and senescent neural cells can further contribute to Aβ plaques and cognitive dysfunction in a vicious cycle [17]. By spatially and temporally elucidating neural cell senescence, the mechanisms and roles of specific senescent cells in AD pathology become critical for the development of the precise prevention and senolytic therapy of AD.

## 3. Neural Cell Senescence and Glucose Metabolism

The metabolic pathways of glucose in neural cells mostly depends upon the cell types and the expressions of metabolic-related enzymes [23]. Neurons consume 70–80% of the total energy of the brain with the rest being used by glial cells [24,25]. Neurons in the adult brain mainly rely on an oxidative phosphorylation approach (OXPHOS) to generate ATP and maintain synaptic activity and neurotransmissions [26]. Astrocytes mostly produce lactate through aerobic glycolysis (AG) to provide enough energy for neurons. Thus, the metabolic changes of neurons and glial cells play an important part in neural cell senescence and AD.

### 3.1. Neuron Senescence and Glucose Metabolism

The balance and stability of the brain function depend on the cooperation of multiple neural cells, such as neurons, astrocytes, and microglia [27]. The conventional concept holds that senescence is a unique feature of replicating cells [28]. Nonetheless, a few researchers have observed that postmitotic neurons also exhibit senescent features with abnormal energy metabolism in aging or neurodegenerative diseases [29]. For example, mature neurons in aged mice brains demonstrated accumulated DNA damage, senescence-associated β-galactosidase (SA-β-Gal) activity, heterochromatinization, and SASP profiles [30,31]. In addition, the SA-β-Gal activity, reactive oxygen species (ROS) production, and NLRP1 inflammasome activation increased with the prolongation of the culture time in hippocampal neurons in vitro [32,33]. Other in vitro studies have indicated additional senescent phenotypes in neurons, such as p53-mediated SASPs, as well as mitochondrial dysfunction [34,35], thus enriching our knowledge of aging.

**Figure 2:**
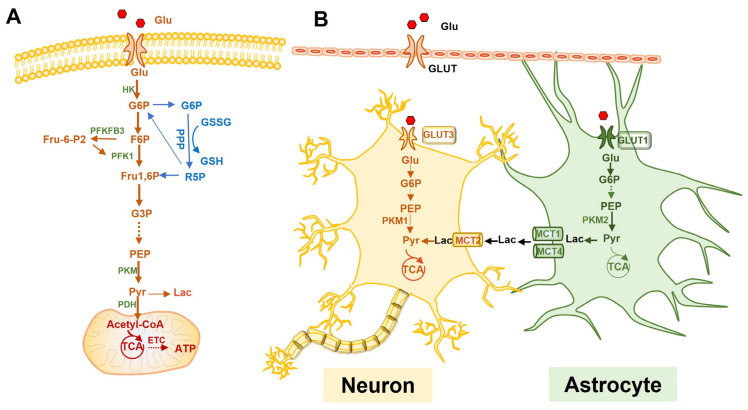
Glucose metabolism and the astrocyte-neuron lactate shuttle (ANLS) hypothesis. (**A**): The metabolic pathway of glucose in neural cells. (**B**): The ANLS hypothesis. Glucose enters astrocytes via GLUT1, which transports to neurons via GLUT3. Astrocytes usually perform aerobic glycolysis to convert glucose to lactate, which is exported outside the cell via monocarboxylate transporter protein 1 or 4 (MCT1/4) and transported to neurons via MCT2. Lactate is then converted to pyruvate in neurons, which is used to promote OXPHOS in the mitochondria. Glu, glucose; GLUT, glucose transporter; G6P, glucose-6-phosphate; F6P, fructose-6-phosphate; Fru1,6P, fructose 1,6-bisphosphate; HK, hexokinase; PFKFB3, phosphofructokinase-2/fructose-2,6-bisphosphatase 3; F-2,6-P, fructose-2,6-diphosphate; PFK1, fructose-phosphate kinase-1; PPP, pentose phosphorylation; TCA, tricarboxylic acid cycle; NADPH, nicotinamide adenine dinucleotide phosphate; PKM1, pyruvate kinase 1; PKM2, pyruvate kinase 2; PDK4, pyruvate dehydrogenase 4; PDH, pyruvate dehydrogenase; Pyr, pyruvate; Lac, lactate; MCT, monocarboxylate transporter; ETC, electron transport chain.

Neurons mainly rely on oxidative metabolism, using glucose as an energy source and using OXPHOS to provide sufficient energy for synaptic transmissions and neuronal function maintenance [26]. When the demand for energy increases, lactate and ketone bodies are also applied as substrates. Compared with other transporters, glucose transporter 3 (GLUT3), located in axons and dendrites, has a higher glucose binding affinity and a greater transport capacity to ensure the preferential use of glucose (Glu) by neurons [36]. Under normal conditions, glucose-6-phosphate (G-6-P) is formed by the action of hexokinase (HK) when glucose enters neurons. Due to the continuous degradation of phosphofructokinase-2/fructose-2,6-bisphosphatase 3 (PFKFB3) in neurons by proteasomes, PFKFB3 is unable to catalyze fructose-2,6-diphosphate (F-2,6-P) and activate fructose-phosphate kinase-1 (PFK1). Thus, G-6-P mostly generates pyruvate through pentose phosphorylation (PPP) and enters the tricarboxylic acid (TCA) cycle and electron transport chain (ETC) [37] (Figure 2A). The nicotinamide adenine dinucleotide phosphate (NADPH) produced by PPP is used for the reutilization of glutathione, thus eliminating the oxygen free radicals produced by neuronal oxidation and avoiding neuronal oxidative damage [38].

On the other hand, senescent neurons exhibit glucose metabolism dysfunctions, such as insulin resistance, glucose transport disturbance, and mitochondrial dysfunction, during the aging process. A recent study using quantitative mass spectrometry-based proteomics, immunofluorescence, and qPCR showed elevated glycogen metabolism in aged hippocampal neurons, indicating that aging increases the capability of neurons to oxidize glucose in glycolysis [39]. Another group reported that lactate dehydrogenase (LDH) was highly expressed in senescent neurons, which catalyze pyruvate to lactate, and the downregulation of LDH in neurons delayed age-related neurodegeneration in Drosophila melanogaster [40]. Taguchi et al. found that a decreased cerebral glucose uptake was strongly associated with a reduced expression of insulin-sensitive glucose transporters [41]. The value of the brain glucose uptake in 24-month-old rats was significantly lower than that of 6-month-old rats and the expression of GLUT3 and GLUT4 in neurons significantly decreased with age [42]. Boumezbeur et al. found that neuronal mitochondrial metabolism was reduced by approximately 30% in older compared with younger subjects [43]. Taken together, these findings support the idea that neurons undergo cellular senescence and exhibit similar features to other cells that are capable of proliferating and abnormal glucose metabolism further affects the function of senescent neurons.

### 3.2. Astroglial Senescence and Glucose Metabolism

**Figure 3:**
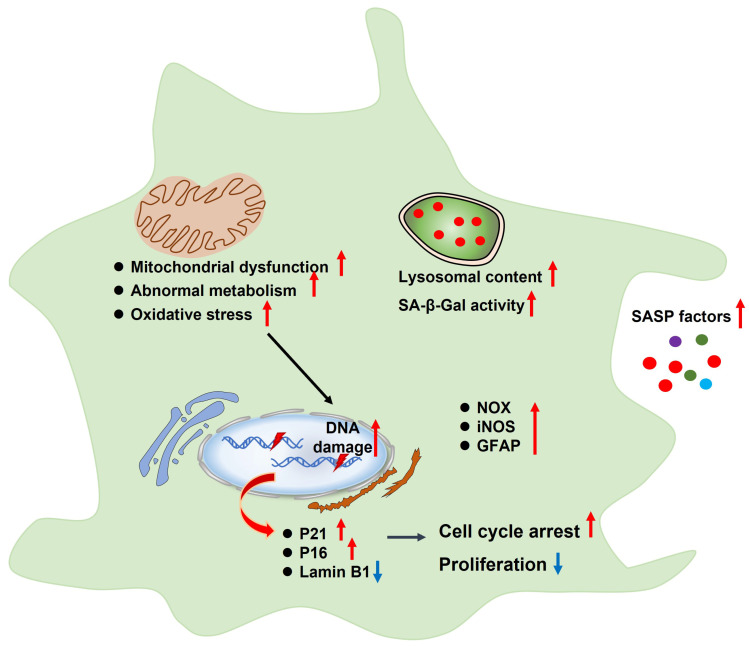
Characteristics of senescent astrocytes, such as cell cycle arrest and decreased proliferation, which are accompanied by mitochondrial dysfunction, abnormal metabolism, increased lysosomal content, SA-β-Gal activity, oxidative stress, DNA damage, senescence-associated gene expression, and SASP release. SASP, senescence-associated secretory phenotype; SA-β-Gal, senescence-associated β-galactosidase; NOX, NADPH oxidase; iNOS, inducible NO synthase; GFAP, glial fibrillary acidic protein.

Astrocytes are the most abundant population in the brain. Previous studies have shown that cultured astrocytes displayed senescence-related phenotypes (Figure 3), such as cell cycle arrest and decreased proliferation, which were accompanied by mitochondrial dysfunction, abnormal metabolism, increased lysosomal content, SA-β-Gal activity, oxidative stress, DNA damage, senescence-associated gene expression, and SASP release [44,45]. Moreover, aged or AD brain exhibited senescent astrocytes and abnormal astrocyte function [13]. Limbad et al., found that senescent astrocytes aggravated glutamate toxicity in cortical neurons [46]. Thus, astrocytes are pivotal for maintaining ion homeostasis and synaptic activity as well as suppressing oxidative stress and providing a unique energy supply for neurons [47].

Astrocytes and neurons are complementary in the utilization of glucose via different metabolic pathways. Unlike neurons dominated by OXPHOS, astrocytes rely on glycolysis to produce energy [48]. High levels of PFKFB3 in astrocytes activate PFK to facilitate glycolysis via F-2,6-P transmutation and the highly enriched pyruvate kinase 2 (PKM2) continuously upregulates the glycolytic flux. Pyruvate dehydrogenase kinase 4 (PDK4), which is highly expressed in astrocytes, can inactivate pyruvate dehydrogenase (PDH), prevent pyruvate from entering the TCA, and ultimately allow glucose metabolism mainly through glycolysis to produce lactate (Figure 2A). In addition, astrocytes are the only cell type that can store glycogen in the brain [49]. G-6-P generates glycogen under the effect of glycogen synthase (GS), which is converted to pyruvate and lactate (Lac) by glycogen phosphorylase. Lactate generated from glycolysis or glycogenolysis in astrocytes is then transferred outside through low-affinity monocarboxylate transporters (MCT) 1 and MCT4 and enters the neurons through high-affinity MCT2 where it is transformed into pyruvate for OXPHOS [50]. This process is called the astrocyte-neuron lactate shuttle (ANLS) [23,51] (Figure 2B). Astrocytic metabolism shifts from glycolysis to mitochondrial oxidative metabolism with aging, showing a decreased mitochondrial activity [52]. Souza et al. found that astrocytes in aged rats exhibited a reduced glucose uptake and a decreased GLUT1 expression [53]. In addition, senescent astrocytes showed an age-dependent increase in oxygen consumption and mitochondrial aerobic metabolism, allowing the astrocytes to switch from neurotrophic to neurotoxic [54]. However, this study did not discuss the increased mitochondrial efficiency; higher rates of oxygen consumption may be also attributed to a leaky mitochondrial membrane and the sub-optimal performance of mitochondrial enzymes. Furthermore, senescent astrocytes created an environment permissive to synapse elimination and neuronal damage, potentially confirming that the abnormal function of senescent astrocytes due to metabolic shifts contributes to a cognitive decline [55]. Overall, the notion of a metabolic shift from glycolysis to mitochondrial metabolism in senescent astrocytes is not an agreed concept. Nevertheless, astrocyte senescence is an emergent field and further research needs to be performed to examine the glucose metabolism of senescent astrocytes.

### 3.3. Microglial Senescence and Glucose Metabolism

Microglial cells, accounting for 5–10% of the central nervous system (CNS) cells, are the most important phagocytes and resident immune cells in the CNS [56,57]. They also play an important role in maintaining brain homeostasis and defense against pathogens and toxins [58,59,60]. Accumulating studies have showed that ageing microglia demonstrated senescent signatures both in vitro and in vivo. For instance, Flanary et al. indicated that cultured rat microglial cells exhibited replicative senescence and were susceptible to telomere shortening in vitro [61]. Subsequently, similar findings were observed in aged rats and AD patients that microglia exhibited significantly decreased telomerase activity [62]. Moreover, lipopolysaccharides (LPSs) and Aβ induced the senescence of cultured BV2 microglial cells with senescence-like phenotypes, such as growth arrest, increased SA-β-Gal activity, and pro-inflammatory cytokine production, suggesting that senescent microglia are involved in the pathogenesis of AD [63,64].

Studies have shown that the function of microglia is closely related to glucose metabolism [65]. Microglia express all the key enzymes of glucose metabolism, including GLUT1 and GLUT3 [66]. The polarization status of microglia affects the glucose metabolic pathway either through glycolysis or OXPHOS [67]. In the “resting” state, or M2 type, microglia mainly rely on OXPHOS for energy demand. When switching to a pro-inflammatory M1 type, the metabolic pattern changes from OXPHOS to glycolysis and is accompanied by an increased glucose uptake and lactate production. Microglia display senescence-related manifestations in the aging brain [68]. When microglia are exposed to inflammatory stimuli, such as IFN-γ or LPS combined with Aβ, the glucose metabolism is converted from OXPHOS to glycolysis [69,70]. The pro-inflammatory activation of microglia is a hallmark of AD and this process involves a switch from OXPHOS toward glycolysis, as demonstrated in APP/PS1 (amyloid precursor protein/presenilin 1 gene) double transgenic mice or 5xFAD mice models [69,71,72]. Furthermore, PKM2, PFKFB3, and IL-1β are highly expressed in microglia of aged mice, which could induce a shift to glycolysis as demonstrated by a decreased oxygen consumption rate (OCR) and an increased extracellular acidification rate (ECAR) [72,73]. All these results suggest that senescent microglia with a metabolic switch play a vital role in the occurrence of AD.

## 4. AD and Abnormal Glucose Metabolism

Neurons are particularly sensitive to energy fluctuations, which lead to age-related diseases, such as AD [74]. An increasing number of studies have shown that the onset and progression of AD are closely linked to glucose metabolism dysfunction in the brain [75,76]. Abnormal energy metabolism in the brain of AD patients precedes Aβ deposition and Tau protein phosphorylation [7]. Aβ-induced neurotoxicity is involved in neuronal energy deficits [77]. Therefore, AD is also a metabolic disease [78]. Revealing the distinct role of glucose metabolism in the pathogenesis and interventions of AD, such as insulin signaling as well as glucose and lactate metabolism, is of great significance for further translational studies.

### 4.1. Insulin Signaling Pathway and AD

The uptake and utilization of glucose by neurons are also affected by insulin. Insulin is a polypeptide hormone secreted by pancreatic beta cells that control the amount of glucose in the bloodstream at a given moment as well as glucose metabolism in the brain [79]. Insulin signaling and its downstream targets affect neuronal activity and synaptic plasticity [80]. Thus, an alteration to insulin signaling can lead to metabolic impairment, cognitive decline, and even the onset and development of AD [81]. Studies have shown that Aβ deposition and Tau protein phosphorylation are associated with abnormal insulin receptor (IR) signaling cascades [82]. Under normal physiological conditions, insulin binds to IR and triggers the phosphorylation of insulin receptor substrate 1 (IRS-1), which in turn activates PI3K and a series of cellular responses, including neuronal growth and synaptic plasticity [81,83,84]. However, insulin resistance could inhibit the phosphorylation of IRS-1 by activating JNK kinase and can result in reduced glucose metabolism, which ultimately leads to memory impairment [85]. Therefore, AD, also regarded as “type 3 diabetes”, is closely related to insulin resistance [86]. In addition, insulin resistance and JNK kinase inhibiting the PI3K/AKT signaling pathway result in the slowdown of neuronal growth as well as impaired synaptic plasticity and cognition deficits [83]. Moreover, the insulin-mediated PI3K/AKT cascade increases insulin-degrading enzymes (IDE); insulin resistance prevents IDE from degrading insulin and Aβ, ultimately leading to Aβ depositions [87,88]. Thus, insulin resistance and altered insulin signaling can induce metabolic abnormalities and the occurrence of AD. Targeting the insulin signaling pathway may offer new strategies for AD prevention and treatment. It was reported that apomorphine improved memory function, reduced insulin resistance, and increased IDE levels in 3×Tg-AD mice by stimulating insulin signaling [89]. Recently, intranasal insulin administration was proven to activate the IRS-1-PI3K-AKT-GSK3β insulin signaling pathway and restored cognitive function in streptozotocin-induced AD mice by reversing insulin resistance [90].

### 4.2. Glucose Metabolism and AD

Glucose is the main energy source for the adult brain. In the resting state, the human brain accounts for 2% of the total body weight, but consumes about 20% of glucose-derived energy to meet the demand [75]. Neurons, with the highest demand of energy, cannot produce and store glucose, but need glucose transporter proteins to transport glucose from the periphery across the blood–brain barrier to the hippocampus and cortex [91]. It is then supplied through OXPHOS, glycolysis, TCA, and PPP to produce ATP [92]. Cognitive impairments in AD are associated with abnormalities in brain glucose utilization as well as glycolysis and OXPHOS metabolism [93,94]. Altered glucose transporter proteins and insulin resistance are two distinct metabolic features of AD patients [95,96,97,98,99]. GLUT1 and GLUT3 are also significantly reduced in the brains of AD patients [100,101]. Altered glucose metabolism usually precedes Aβ deposition, which indicates this early event before the onset of the disease and further facilitates Aβ accumulation and abnormal Tau hyperphosphorylation [95]. Pagani et al. demonstrated that a progressive decrease in the brain glucose metabolism rate examined by FDG-PET could be a marker for a mild cognitive impairment [102]. In 2020, Johnson et al. reported that an abnormal expression of metabolic-related proteins in astrocytes and microglia was strongly associated with AD based on a large-scale proteomic analysis [103]. Several drugs targeting glucose metabolism have been used on AD models. Liraglutide, a glucagon-like peptide-1 (GLP-1) analogue, could increase the transport capacity of the glucose transport carriers and prevent the development of AD [104]. Mullein increased the expression of GLUT3 and GLUT4 in the hippocampus, promoted glucose transport, and enhanced learning and memory in rats [105]. Thus, these studies suggest that impaired glucose metabolism disrupted glucose homeostasis. Remodulating glucose metabolism may be a novel strategy for the intervention and treatment of AD.

### 4.3. Lactate Metabolism and AD

Lactate has long been considered to be a metabolic byproduct or waste. However, recent studies have shown that lactate can act as an energy substrate [106,107]. Astrocytes use lactate generated from AG as energy for neurons [108]. The concentration of lactate is much higher in astrocytes than in neurons and lactate needs to be transported from astrocytes to neurons via the MCT to maintain the dynamic balance of lactate metabolism and regulate long-term memory [51,109,110,111,112]. Zhang et al. found that APP/PS1 mice showed a significant reduction in lactate and monocarboxylate transporters [113]. PTEN knockout mice developed learning and memory deficits accompanied by a lactate accumulation whereas the overexpression of MCT1 or the deletion of Akt1 in the cerebrovascular region reduced the lactate levels and rescued hippocampal degeneration [114], suggesting that lactate homeostasis is essential for neurogenesis and cognition in the hippocampus [48,115]. Liguori et al. found that lower lactate levels in the cerebrospinal fluid of AD patients were associated with higher Aβ and Tau levels [116]. An intrahippocampal infusion of lactate enhanced memory in rats and this effect was inhibited by an MCT2 inhibitor [109]. Curcumin increased lactate and MCT2 protein levels, which in turn improved spatial learning and memory in APP/PS1 mice [117]. Interestingly, lactate was also found to be a signaling molecule that could induce histone lactylation, thereby facilitating the restoration of homeostasis in brain tissues and participating in memory formation and neuroprotection [48,118,119,120]. Interestingly, Pan et al. first observed elevated histone lactylation in brain samples from both 5×FAD mice and AD patients, identifying a lactate-dependent histone modification, histone 4 lysine 12 (H4K12la). This was particularly upregulated in Aβ plaque-adjacent microglia, enriched by the promoters of the glycolytic genes, and activated transcription, thereby increasing the glycolytic activity. This glycolysis/H4K12la/PKM2-positive feedback loop led to a microglia homeostasis imbalance and neuroinflammation and then exacerbated the neuropathy of AD [72]. This in-depth study threw light on the mechanism of microglial lactate metabolic dysfunction and the novel epigenetic regulation of AD development, which is meaningful for innovative translational drug discoveries for AD. Above all, lactate metabolism and histone lactylation in the brain closely participate in the pathogenesis of AD, which may be a novel target for the early diagnosis of and interventions for AD.

## 5. Glucose Metabolic Reprogramming for Potential AD Therapy

So far, the main clinical drugs approved by Food and Drug Administration (FDA) for AD treatment are cholinesterase inhibitors (tacrine, donepezil, carboplatin, and galantamine) and memantine [121]. These drugs are limited in their ability to ameliorate AD symptoms and cannot stop the progression of AD or rescue it completely [122,123,124]. Most drugs targeting Aβ and Tau phosphorylation have failed in clinical trials [125,126]. On 7 June 2021, the FDA approved aducanumab as the first monoclonal antibody drug, which activates the immune system to clear deposited plaques and mechanically prevents AD progression [127]. However, this conditional approval by the FDA is quite controversial and the final result of this clinical trial is still pending [127]. Therefore, it is urgent to develop novel drugs for the treatment of AD by using innovative strategies. As mentioned above, metabolic abnormalities play an important role in the pathogenesis of AD. Most importantly, it is glia, not neurons, that are affected early by brain aging [128]. A chronic impairment in microglia–neuron crosstalk may lead to the permanent loss of synaptic and neuronal functions in AD [129]. These studies suggest that metabolic reprogramming targeting glial cells (microglia or astrocytes) may be a novel strategy for AD intervention and treatment (Table 1).

### 5.1. Microglial Metabolic Reprogramming and AD Intervention

Microglia, the primary innate immune cells and potent immune effector cells of the brain, are able to perform a broad range of functions by changing their energy metabolism patterns from OXPHOS to AG depending on the different state of the cellular phenotypes [141,142,143]. Although glycolysis produces much less ATP than OXPHOS, its reaction rate is 10–100 times faster than OXPHOS, allowing it to meet the high energy demands of activities, such as stress, cell proliferation, migration, cytokine secretion, and phagocytosis [130]. The metabolic switch from mitochondrial OXPHOS to glycolysis has been shown in bacterial, LPS, or interferon-activated M1 microglia and macrophages [142,144,145]. Microglial glycolysis plays a crucial role in AD progression [146,147,148].

Emerging studies have revealed a chronic or acute exposure of Aβ-derived microglial metabolism from OXPHOS to AG via the mTOR/AKT/HIF-1a pathway [130,131]. Thus, the metabolic reprogramming of microglia may have beneficial effects for AD. For example, Capsaicin promoted glucose metabolism, reduced Aβ pathology, and reversed memory deficits in APP/PS1 mouse models [131]. Sodium rutin contributed a metabolic switch from AG to OXPHOS to provide enough ATP for microglia to clear Aβ plaques, suggesting its promising candidacy for AD prevention by reprograming the microglia metabolic approach [132]. A recombinant interferon-γ protein treatment also restored microglial phagocytic activity by reversing its defective glycolytic metabolism in 5×FAD mice [130]. Furthermore, the pharmacologic inhibition of PKM2 by shikonin or compound 3K attenuated microglial activation and the microglia-specific ablation of PKM2 disrupted the glycolysis/H4K12la/PKM2-positive feedback loop and improved spatial learning and memory in AD mice [72]. Collectively, these findings suggest that microglial metabolic reprogramming is critical to its function in AD, which may be an alternative and promising therapeutic approach for AD intervention.

### 5.2. Astroglial Metabolic Reprogramming and AD Intervention

Astrocytes, the most abundant glial cells in the brain, have a higher affinity to glucose uptake and primarily use glycolysis to produce lactate for neurons [149,150]. Recent studies have demonstrated that abnormal AG in astrocytes is a primary early event and dominates in the early phase of AD [95,151]. An increased hippocampal astroglial metabolism is associated with aging and memory deficits, suggesting that boosting astroglial metabolic activity may delay a cognitive decline [152]. Aberrant glycolysis-derived L-serine production in astrocytes contributed to an impaired neuronal function whereas a dietary supplementation of L-serine rescued cognitive deficits in AD mice [133]. Ganglioside GM1 could enhance astrocytic glycolysis and neuronal mitochondrial activity shown by an increasing glucose uptake and lactate secretion [134]. Evidently, insulin and insulin signaling pathways in astrocytes have received much attention due to their important role in brain glucose uptake and the maintenance of brain glucose homeostasis [153,154,155]. In addition, incretin glucagon-like peptide 1 and the glucose-dependent insulinotropic polypeptide (GLP-1/GIP) receptor agonist could alleviate the cognitive decline in AD mice by activating the insulin signaling pathways to maintain the glucose metabolism of astrocytes [135]. Of note, a traditional Chinese medicine named Shen-Zhi-Ling oral liquid exerted neuroprotective effects through the activation of the insulin signaling pathway and improved brain glucose metabolism in early AD [136]. Water extracts of *Agrimonia pilosa Ledeb* (APL), *Cinnamomum cassia Blume* (CCB), and *Lonicera japonica Thunb* (LJT) protected against cognitive dysfunction and glucose dysregulation by reducing neuroinflammation and hippocampal insulin resistance in Aβ-induced rats [137]. Collectively, targeting the metabolic reprogramming of astrocytes to reshape the cell viability and brain function may be a unique and effective therapeutic approach for AD.

### 5.3. Diet, Exercise, and AD Intervention

As mentioned above, glucose intolerance, insulin resistance, and other risk factors for the occurrence of AD are strongly associated with the progression of AD [156]. Studies of drugs that could enhance insulin sensitivity were explored. For example, metformin can reverse Aβ-induced metabolic defects in favor of AD [157,158]. Non-pharmacological therapies, such as dietary interventions and physical exercise, have also been widely investigated to intervene in AD by improving insulin sensitivity [159,160]. In recent years, other lifestyle changes, such as caloric restriction, ketogenic diet, and intermittent fasting, all reduced the neuropathological features of AD [161,162]. Ketoester supplementation in the diet was shown to effectively reduce Aβ and Tau phosphorylation and thus improved the cognitive function of AD mice [163,164]. Intermittent fasting improved memory function by enhancing hippocampal insulin signaling and inhibiting Aβ deposition in AD rats [138].

On the other hand, appropriate exercise could also enhance neuroplasticity and comprehensively improve cognitive function. ROS, Aβ accumulation, and Tau hyperphosphorylation were all reduced [165,166]. Frequent physical exercise could alleviate cognitive deficits by improving cerebral blood flow and metabolism [167,168]. Physical exercise could also improve cognitive function and glucose metabolism by switching the microglia phenotype from M1 to M2 in several AD models [139,169,170]. A three-month aerobic exercise program effectively improved brain energy metabolism by increasing the cerebral ketone transport to promote neurogenesis and cognition in AD patients [140]. Thus, diet and exercise could also contribute to modulating brain glucose metabolism and improving AD cognitive dysfunctions.

## 6. Conclusions and Prospects

The pathophysiology of AD is closely correlated with the senescence of neural cells (neurons, microglia, and astrocytes) and abnormal glucose metabolism. It has become popular to illustrate the pathogenesis of and intervention in AD from a metabolic point of view, such as insulin resistance, glucose metabolism, and lactate metabolism. Metabolic reprogramming targeting glial cells (microglia and astrocytes) is a novel direction for AD intervention and treatment, but there are many undefined mechanisms and limitations. For example, energy metabolism-based drugs, such as GLP-1 analogues and natural extracts (e.g., curcumin, sodium rutin, and shikonin), have shown positive effects on AD. However, their bioavailability is very low and uncertain adverse effects need to be further explored. In addition, both astrocytes and microglia have beneficial and/or detrimental roles in the development of AD, which may be related to the stage of AD progression. Thus, there are major challenges to metabolic reprogramming targeting glial cells, which rely on the time window of intervention and treatment. Further studies are necessary to address these concerns and design glia-targeted therapeutic strategies for AD interventions. There is a long way to go to obtain a deep understanding of the links between glucose metabolism, neural cell senescence, and AD, which will eventually contribute to the early diagnosis, effective prevention, and treatment of AD.

## Figures and Tables

**Table 1:** Metabolic reprogramming of glial cells (microglia and astrocytes) in AD.

Drugs/ Therapy	Cell/Animal Models	Inducers	Mode of Action	Consequence	Ref.
IFN-γ	Microglia; 5×FAD mice	Aβ	Boost mTOR-HIF-1a pathway and metabolic reprograming	Increase neuronal survival and cognitive function	[130]
Capsaicin	APP/PS1 mice	Aβ1-42 TRPV1-deficient	Rescue impaired microglia metabolism and mTOR signaling	Attenuate memory impairment and amyloid pathology	[131]
Sodium rutin	Microglia; APP/PS1, 5×FAD mice	Aβ	Promote mitochondrial OXPHOS	Ameliorate spatial learning and memory deficits	[132]
L-serine	3×Tg-AD mice	Deficit of serine	Ameliorate serine deficiency caused by glycolysis	Improve spatial memory performance	[133]
Shikonin or compound 3K	Microglia; 5×FAD mice	\	Inhibit PKM2 and then disrupt glycolysis/H4K12la/PKM2-positive feedback loop	Reduce Aβ burden and neuroinflammation; ameliorate cognition	[72]
Ganglioside GM1	Astrocyte; neuron	Ganglioside GM1	Enhance astrocytic glycolysis	Neuroprotective effect	[134]
DA-JC4	Rat	Streptozotocin	Promote re-sensitization of insulin signaling	Reduce level of Tau Improve learning and memory	[135]
Shen-Zhi-Ling oral liquid	SH-SY5Y, APP/PS1 mice	Aβ_42_	Regulate the insulin signal pathway and glucose transporter	Ameliorate cognition	[136]
CCB, LJT, and APL	Rat	Aβ-infused	Activation of insulin signaling	Improve cognitive function	[137]
Intermittent fasting	Ovariectomized rats	Aβ-infused	Suppress insulin resistance and decrease neuroinflammation	Improve memory function	[138]
Treadmill exercise	Rat	Streptozotocin	Prevent oxidative damage	Improve cognition	[139]
Walk	AD patients	/	Increase ketone uptake	Improve cognition	[140]

AD, Alzheimer’s disease; Aβ, amyloid-β; IFN-γ, interferon-γ; APP/PS1, amyloid precursor protein/presenilin 1; 5×FAD, 5×familial Alzheimer’s disease mice; 3×xTg-AD, triple transgenic mice; DA-JC4, novel dual GLP-1/GIP receptor agonist; APL, *Agrimonia pilosa Ledeb*; CCB, *Cinnamomum cassia Blume*; LJT, *Lonicera japonica Thunb*.

## Data Availability

Not applicable.

## Abbreviations

AD, Alzheimer’s disease; Aβ, amyloid-β; NFTs, neurofibrillary tangles; SASP, senescence-associated secretory phenotype; SA-β-Gal, senescence-associated β-galactosidase; OXPHOS, oxidative phosphorylation system; AG, aerobic glycolysis; ROS, reactive oxygen species; Glu, glucose; GLUT, glucose transporter; G-6-P, glucose-6-phosphate; F6P, fructose-6-phosphate; Fru1,6P, fructose 1,6-bisphosphate; HK, hexokinase; PFKFB3, phosphofructokinase-2/fructose-2,6-bisphosphatase 3; F-2,6-P, fructose-2,6-diphosphate; PFK1, fructose-phosphate kinase-1; PPP, pentose phosphorylation; TCA, tricarboxylic acid cycle; NADPH, nicotinamide adenine dinucleotide phosphate; PKM1, pyruvate kinase 1; PKM2, pyruvate kinase 2; PDK4, pyruvate dehydrogenase 4; PDH, pyruvate dehydrogenase; GS, glycogen synthase; Pyr, pyruvate; Lac, lactate; MCT, monocarboxylate transporter; ETC, electron transport chain; LDH, lactate dehydrogenase; ANLS, astrocyte-neuron lactate shuttle; CNS, central nervous system; LPS, lipopolysaccharide; IR, insulin receptor; IRS1, insulin receptor substrate 1; mtDNA, mitochondrial DNA; OCR, oxygen consumption rate; ECAR, extracellular acidification rate; IDE, insulin-degrading enzymes; GLP-1, glucagon-like peptide-1; GIP, glucose-dependent insulinotropic polypeptide; FDA, Food and Drug Administration; APP/PS1, amyloid precursor protein/presenilin 1.
